# Education is power: preserving cognition in the UK biobank

**DOI:** 10.3389/fpubh.2023.1244306

**Published:** 2023-09-28

**Authors:** Benjamin Tari, Morgane Künzi, C. Patrick Pflanz, Vanessa Raymont, Sarah Bauermeister

**Affiliations:** ^1^Department of Psychiatry, University of Oxford, Oxford, United Kingdom; ^2^Centre for the Interdisciplinary Study of Gerontology and Vulnerability, University of Geneva, Geneva, Switzerland

**Keywords:** education, cognition, socioeconomic status, greenspace, coastal distance

## Abstract

**Introduction:**

Dementia is a debilitating syndrome characterized by the gradual loss of memory and cognitive function. Although there are currently limited, largely symptomatic treatments for the diseases that can lead to dementia, its onset may be prevented by identifying and modifying relevant life style risk factors. Commonly described modifiable risk factors include diet, physical inactivity, and educational attainment. Importantly, however, to maximize the utility of our understanding of these risk factors, tangible and meaningful changes to policy must also be addressed.

**Objectives:**

Here, we aim to identify the mechanism(s) by which educational attainment influences cognition.

**Methods:**

We investigated data from 502,357 individuals (*M_age_* = 56.53, *SD_age_* = 8.09, 54.40% female) from the UK Biobank cohort via Structural Equation Modelling to illustrate links between predictor variables (i.e., Townsend Deprivation Index, coastal distance, greenspace, years of education), covariates (i.e., participant age) and cognitive function as outcome variables (i.e., pairs-matching, trail-making task B, fluid intelligence).

**Results:**

Our model demonstrated that higher education was associated with better cognitive performance (ps < 0.001), and this relationship was mediated by indices of deprivation, and coastal distance.

**Conclusion:**

Accordingly, our model evinces the mediating effect of socioeconomic and environmental factors on the relationship between years of education and cognitive function. These results further demonstrate the utility and necessity of adapting public policy to encourage equitable access to education and other supports in deprived areas.

## 1. Introduction

Dementia characterizes a debilitating syndrome involving cognitive decline and loss of memory severe enough to hamper independent, daily functioning (1). Globally, dementia is a leading cause of death and imparts immense economic, societal and personal burdens (2), with currently limited treatment options available. Moreover, as the global population ages beyond 65 years, the incidence of dementia cases is expected to surpass 152 million by the year 2050 (3), and the need to find means to prevent, diagnose and treat dementia becomes even more pressing. Assessing, understanding, and modifying dementia risk factors which precipitate disease onset is a critical step in prevention. Risk factors include poor diet, smoking and alcohol consumption, physical inactivity, depression and lack of education; in combination, these factors may contribute to up to 40% of known dementias (4). In order to maximize the management of relevant risk factors, tangible and meaningful changes to policy must be implemented to support personal mitigation efforts. For example, policies need to narrow the gap between socioeconomic classes to reduce undue stress and improve mental health (5, 6). Providing more equitable access to education for those living in underserviced/impoverished communities is one way to achieve this.

Education is one important modifiable risk factor and evidence suggests that disparities in access to education can encumber disadvantaged individuals. In developed countries, educational attainment has long been presumed to be associated with improved physical (7) and mental health (8), and is highly related to one’s employability, income, and overall individual adulthood socioeconomic status (SES). Children belonging to low SES groups are less likely to develop fundamental reading skills (9), have a lower level of baseline cognitive performance (10, 11), are less likely to have access to learning materials in the home (12), and are more likely to accrue higher-than-average student dept. (13). This combination of factors hinders their ability to be educated to the same degree as high-SES individuals, and may lower their chances of improving their SES in the future; perpetuating the cycle of inequality. Interestingly, the literature regarding the *protective* benefits of education on cognition is mixed depending on whether the focus is on the level or change in cognition (14). That is, higher education has been shown to have no effect on the speed of cognitive decay due to normal aging in some populations (15), whereas Fletcher and colleagues (16) demonstrated that in genotyped siblings, higher educational attainment was associated with higher cognitive scores. The protective benefits seen in the latter may be attributed to higher cognitive reserve accrued, as a result of more education (17, 18). Accordingly, the literature outlined above provides evidence for the need for more equitable access to education and the benefits education may have on one’s cognitive status, as well as one’s mental and physical health.

Moreover, the environment in which individuals live and work influences individuals’ health status. Built environment includes factors such as housing structure and architecture, environmental quality, walkability and green/blue space (19), as well as measures of population density and pollution (20), which differs between urban and more natural rural/coastal environments. Previous research has identified that a higher percentage of greenness is associated with lower risks of psychological distress (19), promotes active daily living, reduces active stress levels, and provides areas for therapeutic healing (21). Indeed, recent research has found that individuals living in greener environments have been found to be less likely to suffer from depression (22) and possess better cardiovascular health (23). Previous work has also proposed that children who live in greener environments are more likely to possess more highly developed cognitive functions (24). However, other work does not support this finding, and suggests that greenspace neither protects nor promotes cognitive development (25) or mental health (26). With regards to coastal distance, the literature is again varied. Similar to those investigations exploring greenspace, Gascon et al. (27) present evidence suggesting there is a positive association between living close to water and improved mental health, well-being, and an increased likelihood of engaging in physical activity. Nutsford et al. (28) also provide evidence that available blue space (e.g., being closer to a coastline) facilitates social interaction and acts to preserve mental health via inherent therapeutic properties. In contrast, in some populations the proximity to water can have negative effects on mental health outcomes. Helbich et al. (29) demonstrated that although living close to inland blue spaces imparted a protective benefit to the mental health of a Dutch cohort of 105,398 individuals, living nearer to a coastline had the opposite effect. Indeed, women, but not men, who lived closer to the Dutch coastline were more likely to commit suicide than those living inland.

The studies included above are a collection of reviews (9, 14, 17, 18, 27), and primary cross-sectional and longitudinal analyses which make use of linear, logistic, multi-level, genomic, and/or structural equation modelling techniques to describe large cohort data (7, 8, 10–13, 15, 16, 19–26, 28, 29). Taken together, the current body of literature provides only mixed results for how education, individual-specific deprivation (i.e., separate contributors of SES), and built environment interact to support or hinder cognitive and mental health outcomes. Moreover, very little has been done in terms of explicitly assessing potential direct and indirect links between these aforementioned variables. That is, the potential mechanism(s) regarding how educational attainment may influence cognitive function via measures of deprivation and built environment is as yet un-examined. To our knowledge, the following investigation is the first to examine this relationship using data from the UK Biobank cohort. We hypothesized that education would have positive effects on cognition; however, this relationship would be better explained by some mediation via metrics of deprivation and built environment.

## 2. Materials and methods

### 2.1. Participants

Data from 502,357 individuals (*M_age_* = 56.53, SD*_age_* = 8.09) from the UK Biobank cohort (30) were included in this project (see Table 1 for more detail). The UK Biobank study received ethical approval from the UK Biobank Research Ethics Committee (approval letter dated 17, June 2011: Ref 11/NW/0382) and was conducted in accordance with the Declaration of Helsinki. All participants gave informed, written consent.

**Table 1:** Participant characteristics, cognitive performance, SES, and built environment.

Characteristics	*N*		
Age	502,357	*M* (SD)	56.53 (8.09)
Sex	502,360	% Female	54.40%
Education	495,642	*M* (SD)	18.11 (2.77)
*Cognitive function*			
Fluid Intelligence	123,579	M(SD)	6.41(2.06)
Trail-making task-B	103,998	M(SD)	66.81(25.75)
Pairs-matching task	118,495	M(SD)	4.20(3.12)
*Deprivation*			
Townsend deprivation index	501,734	M(SD)	−1.29(3.09)
*Built environment*			
Coastal distance (km)	497,397	M(SD)	41.64(27.71)
Percentage greenspace	440,736	M(SD)	35.27(23.22)

Uncorrected group means and standard deviations [*M*(*SD*)] for age (in years), years of education, Townsend Deprivation Index score, percentage of greenspace, coastal distance, fluid intelligence score, trail-making task-B duration, and number of incorrect pairs-matching trials. Note that we also present the % of females in our sample (*N*).

### 2.2. Predictor variables

#### 2.2.1. Built environment, deprivation, and education

Our analyses included three predictor variables: built environment, deprivation and education. Built environment was estimated by participants’ distance to the coastline and the percentage of greenspace around where they lived. The Townsend Deprivation Index (TDI) was used to quantify individual levels of deprivation. The TDI variable used here is a standardized individual rating of deprivation which in and of itself reflects one’s “real” living conditions according to geographic constraints and not simply a rating of poverty (31). The TDI incorporates common measures normally used as a proxy of SES such as of unemployment, car-ownership, home-ownership and home overcrowding, but excludes education (31). Higher values on this index indicate higher levels of deprivation and lower SES. Finally, years of education was separately calculated via an algorithm which imputed years of education to the missing values of the “age completed full time education” variable based on the “qualifications” variable. The inclusion of both TDI and education-related variables allows us to understand the degree to which these components commonly assumed to contribute to SES influence each other and cognitive function. Participant age was included in our analysis as a co-variate. These data were collected between 2006 and 2010.

### 2.3. Outcome variables

#### 2.3.1. Cognitive function

Cognitive function was estimated via three separate tests: the 6-pair pairs-matching test (PM6), the trail making task-B (TMTB), and an examination of fluid intelligence (FI). These tests assessed various aspects of cognition including memory, executive function, and abstract reasoning, respectively (see UK Biobank data showcase for more detail; https://biobank.ndph.ox.ac.uk/showcase/) and were included due to their sensitivity to cognitive decline/disruption over the lifespan (32). The cognitive variables used here are comprised of data taken between 2014 and 2015 and therefore the volume of collected data may differ from predictor variables according to rates of attrition.

### 2.4. Statistical analyses

#### 2.4.1. Pre-processing

Data were pre-processed and analyzed using Stata SE 17.0 via the Dementias Platform UK (DPUK) Data Portal (33). Participants aged 40–73 were included in our analyses. This relatively large age-range was retained in order to assess the validity of a model which predicts cognitive function across time, rather than in an age-range in which dementia typically occurs (i.e., 60–70 years) (1). We assessed the normality of our variables of interest (see below) and where appropriate, skewed (i.e., *g*_1_ > 1.0) data were log-transformed for normalisation.

#### 2.4.2. Spearman correlations

We employed Spearman correlations on our data to explore associations between measures of deprivation, coastal distance, greenspace, the number of incorrect responses on the PM6, the duration of an alphanumeric path in the TMTB, total FI scores, years of education and age (Table 2). Correlations were Bonferroni corrected and associations were considered significant if *p* < 0.01.

**Table 2:** Correlation matrix for participant cognitive variables.

	Age	Education	TDI	Coast	Green	PM6	TMTB	FI
Age	–	–	–	–	–	–	–	–
Education	−0.13⁑	–	–	–	–	–	–	–
TDI	−0.10⁑	0.002	–	–	–	–	–	–
Coast	0.01	0.02⁑	−0.06⁑	–	–	–	–	–
Green	0.05⁑	−0.06⁑	−0.30⁑	0.05⁑	–	–	–	–
PM6	0.14⁑	−0.04⁑	−0.002	0.01	−0.001	–	–	–
TMTB	0.38⁑	−0.22⁑	0.02⁑	−0.01⁑	0.01	0.18⁑	–	–
FI	−0.10⁑	0.33⁑	−0.03⁑	0.01*	−0.02⁑	−0.12⁑	−0.42⁑	–

Correlation matrix *r* values for all variables of interest with Bonferroni corrected significance levels. TDI, Townsend Deprivation Index; PM6, 6-pair pairs-matching task; TMTB, trail-making task-B; FI, fluid intelligence. Associations marked with * indicate *p* < 0.01; those marked with ⁑ indicate *p* < 0.001. *N* = 88,436.

#### 2.4.3. Structural equation model

We employed a structural equation model (SEM) to assess direct and indirect effects between education, deprivation and built environment on cognition. That is, we aimed to create a single model to assess a possible mechanistic pathway by which our predictor variables may influence cognitive outcomes. Prior to creating our model, simple regressions of the variables of interest were performed to better inform direct and indirect model paths. Our model was estimated using a maximum likelihood with missing values (MLMV) test and we report standardized coefficients and beta values. The MLMV method assumes joint normality and, if present, randomly occurring missing values. The resulting model contains the following variables.

**Figure 1:**
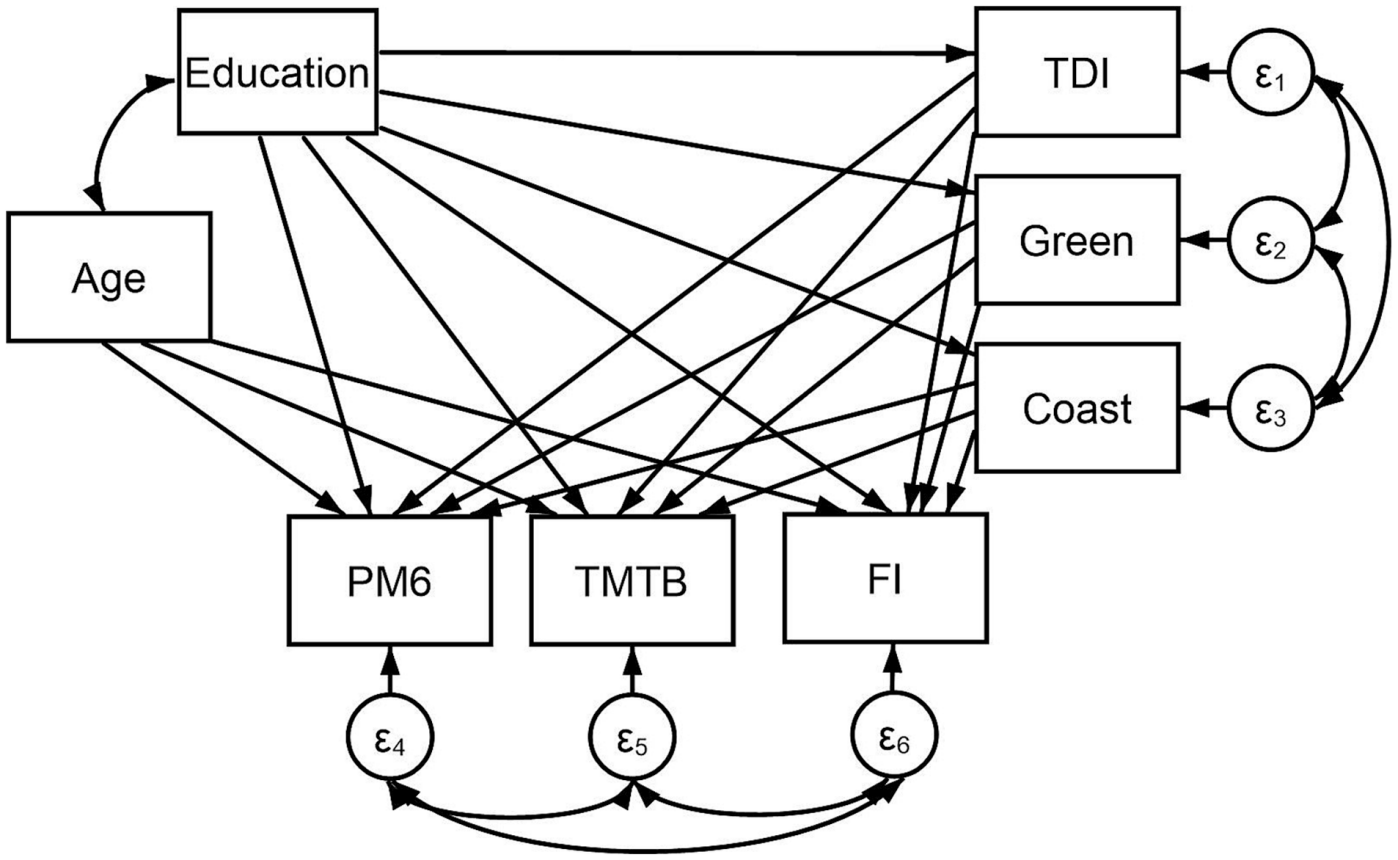
Structural equation model including predictor variables: Education (i.e., imputed years of education), TDI (i.e., Townsend Deprivation Index total score; variable: 22189), Coast (i.e., the distance to the coastline in kilometers; variable: 24508) and Green (i.e., percentage of greenspace within a 300 m buffer area; variable: 24503); and mediator variable: Age (variable: 21022). Paths extend from these variables to three cognitive variables: PM6 (i.e., number of incorrect responses on the pairs-matching task; variable: 20132), TMTB (i.e., the time required to complete the trail-making task-B alphanumeric path; variable: 20157), and FI (i.e., fluid intelligence score; variable: 20191). Covariance links join Age and Education; FI, TMTB, and PM6; and TDI, Green, and Coast. *N* = 502,357.

Cognitive function included incorrect PM6 responses (variable: 20132), the time required to complete the TMTB alphanumeric path (variable: 20157), and a score of FI (variable: 20191). Cognitive variables were allowed to covary. We chose to not represent these variables in a latent construct in order to assess the differential effects of our predictor variables on various aspects of cognition. Predictor variables included education, deprivation, and built environment which were represented by imputed years of education, scores on the TDI (variable: 22189), the distance to the coastline in kilometers (Coast; variable: 24508) and the percentage of greenspace within a 300 m buffer area (Green; variable: 24503), respectively. The three latter variables were connected via covariance links to assess their relationship. As the direction of any association between these variables cannot be confirmed here, we chose to omit direct path links between them. Age (a continuous variable; variable: 21022) was also entered into our SEM to control for any confounds and was linked to Education via a covariance link (Figure 1). Due to the large sample size, effects were deemed significant when *p* < 0.01 (34). For ease of replication, we have included our STATA script in the Supplementary material of this work.

## 3. Results

### 3.1. Participant characteristics

Our sample was on average 56.53 (SD = 8.09) years of age, comprised of mostly females (54.4%) and had completed a mean of 18.11 (SD = 2.77) years of education (Table 1).

### 3.2. Spearman correlations

Our results indicated that years of education was correlated to all cognitive variables (rs > −0.04, ps < 0.001). TDI scores correlated with TMTB times (*r* = 0.02, *p* < 0.001) and with FI (*r* = −0.03, *p* < 0.001), whereas coastal distance and greenspace were correlated only to TMTB times (*r* = −0.01, *p* < 0.001) and FI (*r* = −0.02, *p* < 0.001), respectively. We note also that all the cognitive variables used were correlated to each other (rs > −0.12, ps < 0.001), as were TDI, greenspace and coastal distance (rs > 0.05, *ps* < 0.001). Finally, years of education was correlated to all the variables included in our model (rs > 0.02, ps < 0.001) except TDI (r = 0.002, *p* > 0.99). A full correlation matrix is presented in Table 2.

### 3.3. Structural equation model

#### 3.3.1. Regression paths

Figure 1 demonstrates direct paths extending from each predictor variable (i.e., Education, TDI, Coast, Green) and Age to the cognitive outcome variables (i.e., PM6, TMTB, and FI). Direct links were also included between Education and TDI, Coast, and Green to assess the mediation of the putative relationships between education and various cognitive domains.

#### 3.3.2. Estimation and fit

The model fit was deemed “good” according to accepted standards [e.g., (35)]. The root mean squared error of approximation (RMSEA: differences between predicted and observed outcomes) = 0.06, and the comparative fit index (CFI: metric of the model’s improvement from baseline to proposed iterations) = 0.94.

**Figure 2:**
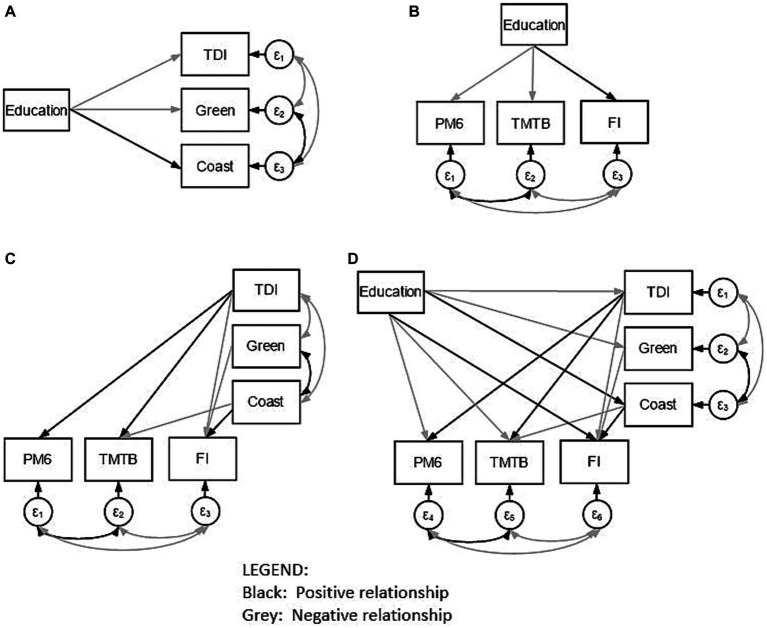
A simplified illustration of our model (D) as well as individual panels illustrating direct and indirect links between predictor and outcome variables as well as covariance links: education and the Townsend Deprivation Index (TDI), greenspace (Green), and coastal distance (Coast) **(A)**; education and scores on the pairs-matching task (PM6), the trail-making task B (TMTB) and fluid intelligence (FI) **(B)**; and Townsend Deprivation Index, greenspace and coastal distance with scores on the pairs-matching task, the trail-making task B and fluid intelligence are also included **(C)**. Note that only statistically significant positive (black arrows) and negative (gray arrows) interactions are presented. The covariate of Age is not included for ease of visualization.

Figure 2 demonstrates only statistically significant path links within our model. In particular, lower education was associated with higher deprivation (β = −0.07, *p* < 0.001), living closer to the coast (β = 0.01, *p* < 0.001) and inhabiting an area with more greenspace (β = −0.01, *p* < 0.001). The covariance links between TDI, coastal distance and greenspace were also statistically significant (β > 0.03, *p* < 0.001) indicating that higher deprivation was associated with living closer to the coast and in greener areas. Moreover, higher education was shown to predict better performance on the PM6 (β = −0.02, *p* < 0.001) and TMTB (β = −0.17, *p* < 0.001), and higher FI (β = 0.32, p < 0.001). Lower deprivation was related to fewer incorrect PM6 responses (β = 0.01, *p* < 0.001), shorter TMTB times (β = 0.08, *p* < 0.001) and greater FI (β = −0.06, *p* < 0.001). Living closer to the coastline was associated with longer TMTB times (β = −0.01, *p* < 0.001) and lower FI (β = 0.01, *p* = 0.003); inhabiting an area with less greenspace was related only to higher FI (β = −0.01, *p* < 0.001). Results demonstrated a partial mediation of the effect of education on cognitive performance by measures of deprivation and built environment. Indeed, β values for PM6, TMTB and FI were attenuated from (−0.07) to (0.01, 0.08, and − 0.06) by TDI. We also see attenuation of β values of the relationship between education and TMBT and FI from (0.01) to (−0.01 and − 0.01, respectively) by coastal distance. Greenspace was not shown to mediate the relationship between education and cognition. Finally, older age was associated with poorer performance on all cognitive variables (βs > −0.09, ps < 0.001). See Table 3 for the full SEM output.

**Table 3:** Structural equation model output.

	Predictor	*β*	*SE*	*z*	*p*	95% CI	
*TDI*							
	Education	−0.07	0.001	−51.22	<0.001*	−0.076	−0.070
*Coast*							
	Education	0.01	0.001	9.15	<0.001*	0.01	0.016
Green							
	Education	−0.01	0.002	−5.23	<0.001*	−0.011	−0.005
*PM6*							
	TDI	0.01	0.003	3.64	<0.001*	0.006	0.019
	Coast	0.006	0.003	1.99	0.046	0.0001	0.012
	Green	−0.002	0.003	−0.53	0.600	−0.008	0.004
	Age	0.13	0.003	43.09	<0.001*	0.124	0.136
	Education	−0.02	0.003	−5.35	<0.001*	−0.023	−0.011
*TMTB*							
	TDI	0.08	0.003	24.97	<0.001*	0.072	0.084
	Coast	−0.01	0.003	−5.31	<0.001*	−0.020	−0.009
	Green	0.002	0.003	0.70	0.485	−0.004	0.008
	Age	0.38	0.003	147.44	<0.001*	0.376	0.387
	Education	−0.17	0.003	−58.59	<0.001*	−0.178	−0.166
*FI*							
	TDI	−0.06	0.003	−21.25	<0.001*	−0.071	−0.059
	Coast	0.01	0.003	2.99	0.003*	0.003	0.013
	Green	−0.01	0.003	−4.27	<0.001*	−0.018	−0.007
	Age	−0.09	0.003	−30.48	<0.001*	−0.091	−0.080
	Education	0.32	0.003	117.56	<0.001*	0.312	0.323

The left most column indicates the variables of interest and include: TDI (i.e., Townsend Deprivation Index), Coast (i.e., the distance to the coastline in kilometers), Green (i.e., percentage of greenspace within a 300 m buffer area), PM6 (i.e., number of incorrect responses on the 6-pair pairs-matching task), TMTB (i.e., the time required to complete the trail-making task-B alphanumeric path) and FI (i.e., fluid intelligence score). Predictor variables indicate those which have direct paths to the variable of interest. The output provides standardized beta (*β*) values, standard error (*SE*), *z* scores, *p* values, and 95% confidence intervals (95% CI). For ease of interpretation, *p*  values marked with * indicate a statistically reliable association: *p* < 0.01. *N* = 502,357.

## 4. Discussion

We sought to explore how education, deprivation and built environment influence cognition in a middle-aged cohort using UK Biobank. The following sections will discuss the links between age and cognition, as well as how education’s influence on various domains of cognition is mediated by measures of deprivation and built environment.

### 4.1. Deprivation and coastal distance mediate the relationship between education and cognitive function

Education is one of the single most important modifiable determinants of health which predicts employment, income, overall SES and well-being (36). Our model demonstrates that higher educational attainment was found to be positively associated with improved cognitive performance. We provide evidence for the association between higher education and fewer incorrect PM6 responses, shorter TMTB durations, and better FI. As expected, these associations were all found to be mediated by individual deprivation levels; however, our model also demonstrated that built environment factors commonly assumed to impart positive health benefits (26, 27) had the opposite effect. Here, the proximity to a coastline did not predict PM6 performance. In contrast, living closer to the coastline was related to longer TMTB durations and worse FI. Similarly, although greenspace percentage was not related to either PM6 or TMTB task performance, living in an area with more greenspace was related to worse FI. We will address these points in turn and will then provide some examples of prospective policy changes which warrant further investigation.

First, in addressing the positive relationship between education and cognition, a parsimonious explanation of our results can be provided via the cognitive reserve hypothesis (27). Cognitive reserve has been proposed to be an amalgamation of individual differences in cognitive processing which allows for one to better cope with normal and/or abnormal processes of aging. For example, higher cognitive reserve has been thought to slow the onset of cognitive decline (18). Indeed, higher levels of education are associated with more cognitive stimulation, as well as better income and more stable work environments commonly associated with higher SES. As well, the combination of more education and the greater opportunities afforded individuals of higher SES (37) likely plays a direct role in the accumulation of psychological resilience (38) and cognitive reserve (39). Together, these factors have been shown to have a protective effect on cognition in mid- and late-life and reduce the likelihood of developing dementia (4, 40). Second, with respect to the mediation of these results by indices of deprivation and built environment, we posit that lower levels of deprivation provide individuals with an assortment of benefits which support cognition in later life, and factors specific to rural and coastal regions of the United Kingdom outweigh the oft-reported benefits of coastal/rural living. Regarding the former, we should acknowledge that these results were obtained in a context of abnormally high educational attainment within our sample. It may be that deprivation exerts its influence on cognition via factors such as reduced access to nutritional and/or health-related resources, and higher levels of chronic stress (41, 42). Indeed, chronic stress related to higher levels of deprivation is likely to dysregulate neuro-endocrine activity (i.e., the release of catecholamines and cortisol via hypothalamic–pituitary–adrenal axis) which may negatively affect the efficiency by which neurons are activated during a given task (43) and subsequently alter performance on higher-level cognitive processes (42, 44). In addressing our second point, recall that our results indicate that living in areas with more greenspace and closer to coastlines have a negative effect on cognitive function. This is interesting as the literature mostly contains evidence which supports the positive association between more greenspace and coastal distance and cognition. Besser (45) demonstrates that most papers reviewed describe a positive relationship which is in line with Gascon et al. (27) who found consistent positive associations with blue space and overall health and well-being. An explanation for contrary results presented here may be that living in extensive green or blue (i.e., rural) areas induce feelings of social isolation (46), increasing stress and reducing cognitive performance (see above). These results also emphasize the need to consider the association between cognition and the built environment in a more inclusive socioeconomic/demographic context (e.g., deprivation and education). To that end, we note that the progression of British students into higher education is markedly lower in coastal and rural regions compared to other urban areas (47), and this would likely be associated with higher levels of deprivation in these areas that contributes to subsequent declines in cognition (see above). Asthana and Gibson (47) further explain how social expenditure is lower in deprived coastal areas compared to similarly deprived inland regions, and that investment in education is highly skewed toward the already best performing UK region (i.e., London). Indeed, a report by the UK Department of Education (48) indicates that educational achievement is considerably lower for disadvantaged individuals living in coastal regions compared to their inland counterparts. The needs of these areas to be serviced in terms of educational opportunity may be overlooked because of the incorrect perception of idyllic rural and coastal environments (49), and perhaps due to the positive benefit assumed to be conveyed by coastal and/or rural living [e.g., (24, 50, 51)]. These benefits may be presumed – perhaps incorrectly – to offset the comparative neglect faced by these regions. Our results do provide further evidence supporting a gap in educational attainment between regions of higher and lower deprivation in the United Kingdom, and the regional specificity in which these gaps exist. This disadvantage will likely translate to less access to resources and greater exposure to occupational hazards and psychological stressors (52, 53), lower cognitive reserve (17, 18) and an increased likelihood of developing non-communicable diseases (54) and dementia (23); however, more research is required to better understand why these results occurred.

### 4.2. Implications

Our results suggest that individuals with higher levels of education are more likely to have easier access to indices of wealth (i.e., a house, a car) and the means by which these indices may be obtained (e.g., a well-paying job). A subsequent implication may be that individuals within a society, and the society as a whole, would be better served to broaden access to means of education, as well as provide more opportunities to become employed. At face value, it may seem that simply providing access to educational resources would be a sufficient solution to provide passive protection to individuals’ cognitive health in later life. Of course, access to resources cannot guarantee that individuals make use of them. What is more, what one individual considers to be a stimulating work environment may differ from another, and that our results demonstrate mediation of this relationship by indices of deprivation and built environment indicates the need for region-specific policies rather than blanket solutions. Providing access to traditional education paths should not be the *only* area of focus for policy-makers. Regarding the negative associations between greenspace, coastal distance and cognition, we must first be clear that we do not advocate for the removal of green areas or forcing development away from coastal regions; research included herein describe the cognitive and mental health benefits of greenspace and coastal zones. Instead, they imply that individuals with a higher level of education are more likely to reside and work in urban areas which may be devoid of these features. As briefly discussed in preceding, and rather than directly alter the environment in which people live, policies should be enacted which indirectly mitigate the negative results shown here. For example, chronic stress may be reduced via greater subsidization of individuals and families in disadvantaged situations. Similarly, increased investment in deprived areas in terms of mental health support or general resources may help to curb feelings of stress and support cognitive health. More research is necessary to better understand these relationships before substantive changes to policy can be suggested.

### 4.3. Limitations and future directions

The above has demonstrated direct and indirect links by which educational attainment supports cognitive function in mid- to late-life. However, we recognize that our work is limited in several methodological aspects which we will outline below. First, years of education does not necessarily predict the *quality* of education received. Of course, many years of poor education would not necessarily impart the same putative benefits as high quality education. Future work should endeavor to model years and quality of education as well as rates of participant literacy to quantify the degree to which either variable predicts cognitive performance. Second, the data used to quantify built environment were not extensive and only examined two commonly assessed variables in this field. Indeed, our model omitted other indicators of built environment such as noise pollution, neighborhood walkability, and environment density. Future work with this cohort should make use of the UKBUMP dataset – a platform with state-of-the-art spatial network analyses to quantify built environments across the United Kingdom (55). We chose to conduct our analyses without this platform in an effort to generalize this model to other cohorts that are without similarly sophisticated built environment composites. Third, our model did not include an index of personal income. This may be perceived as a salient limitation due to the fact it is an oft-used proxy of SES. In addressing this, we note that previous works have outlined how self-reported income, like that in the UK Biobank, may mis-represent individual’s real economic status (56), and is a variable which suffers from under-report (30, 57). We note also, that educational attainment, such as what we have included here, has been previously used as a proxy for one’s income (58). Fourth, this paper has focused primarily on the level of cognitive performance of individuals at a single point in time. Therefore, no implications can be directly drawn from this work regarding how education may directly or indirectly (i.e., via deprivation and/or built environment) influence the rate of cognitive decline. Further longitudinal research is required to elucidate this relationship. Our study is taken advantage of by the use of data from the UK Biobank; a large cohort composed of over 500,000 individuals which contains one of the most detailed datasets in the world. Unfortunately, this sample is skewed in that it is comprised of a single, highly educated, ethnically homogenous British group with distinct cultural, geographic and economic factors which will likely not be generalizable to other global cohorts. As well, whether this model is generalizable even within different counties of the United Kingdom is a question worth exploring, as strong region-specific differences may drive the nationally represented results which we report here.

## 5. Conclusion

Our work demonstrates how educational attainment directly and indirectly mediated cognitive function via individual-specific indices of deprivation and built environment. Accordingly, we provide evidence for the need to improve access to education in deprived/underserviced areas in the United Kingdom, as well as the utility in minimizing the gap in objective material deprivation.

## Data availability statement

Publicly available datasets were analyzed in this study. This data can be found at: https://portal.dementiasplatform.uk/.

## Ethics statement

The studies involving humans were approved by the UK Biobank Research Ethics Committee (approval letter dated 17, June 2011: Ref 11/NW/0382) and was conducted in accordance with the Declaration of Helsinki. The studies were conducted in accordance with the local legislation and institutional requirements. The participants provided their written informed consent to participate in this study.

## Author contributions

BT, MK, CP, VR, and SB designed the study, read, critically revised, and approved the final manuscript. VR and SB provided access to the data. BT, MK, and SB performed the data analysis and interpretation. BT drafted the manuscript with a substantial contribution from all co-authors. VR and SB supervised all stages of this study. All authors contributed to the article and approved the submitted version.
