# Supplementary material for: Education is power: preserving cognition in the UK biobank
Source: Front Public Health. 2023 Sep 28;11:1244306. doi: 10.3389/fpubh.2023.1244306 (PMC10568007; doi:10.3389/fpubh.2023.1244306)
Supplement: Supplementary file 1 [file Table_1.DOCX]

Supplementary Material

Education is power: Preserving cognition in the UK Biobank

Benjamin Tari*, Morgane Künzi, C. Patrick Pflanz, Vanessa Raymont, Sarah Bauermeister

*** Correspondence:** Corresponding Author: benjamin.tari@psych.ox.ac.uk

# Supplementary Data

We include the below STATA script used in analysis as Supplementary Material to facilitate replicability.

**STATA Code**

**** Age***

clonevar age_0_0 = n_21022_0_0

tabstat age_0_0, stats (n mean median sd min max)

drop if age_0_0 < 40

summarize age_0_0, detail

**** Imputed Education***

clonevar education_0_0 = n_6138_0_0

clonevar years_of_edu_0_0 = n_845_0_0

tab1 education_0_0 years_of_edu_0_0

recode years_of_edu_0_0 (-2 = . )

recode years_of_edu_0_0 (-1 = . )

recode years_of_edu_0_0 (-3 = . )

recode education_0_0 (-7 = . )

recode education_0_0 (-3 = . )

**** Generate imputed years of education variable by combining years of education and qualifications***

recode education_0_0 (1 = 21) (2 = 17) (3 = 15) (4 = 15) (5 = 17) (6 = 21), gen (imputed_years_of_education)

gen imp_years_of_education = cond(years_of_edu_0_0 !=. , years_of_edu_0_0, imputed_years_of_education)

tabstat imp_years_of_education, stats (n mean median sd min max)

summarize imp_years_of_education, detailtabstat imp_years_of_education, stats (n mean median sd min max)

summarize imp_years_of_education, detail

**** Sex***

clonevar sex = n_31_0_0

tab sex, freq m

label define sex 0 "Women" 1 "Men", add

label values sex sex

tab sex, freq m

**** Townsend***

clonevar townsend = n_22189_0_0

tabstat townsend, stats (n mean median sd min max)

summarize townsend, detail

histogram townsend, normal

**** Built envir. coast distance***

clonevar coast_0 = n_24508_0_0

tabstat coast_0, stats (n mean median sd min max)

summarize coast_0, detail

histogram coast_0, normal

**** Built envir. greenspace***

clonevar green3_0_0 = n_24503_0_0

tabstat green3_0_0, stats (n mean median sd min max)

summarize green3_0_0, detail

histogram green3_0_0, normal

**** Visual memory***

clonevar vm6_0_1 = n_20132_0_1

tabstat vm6_0_1, stats (n mean median sd min max)

summarize vm6_0_1, detail

histogram vm6_0_1, freq

* ***Visual memory LN + 1 transformation***

generate log_vm6_0_1 = ln((vm6_0_1+1))

histogram log_vm6_0_1, freq

summarize log_vm6_0_1, detail

tabstat log_vm6_0_1, stats (n mean median sd min max)

label variable log_vm6_0_1 "LN + 1 transformation visual memory 6 pairs "

**** Trail Making Test Part B***

clonevar TIME_TMTB_0_0 = n_20157_0_0

tabstat TIME_TMTB_0_0, stats (n mean median sd min max)

summarize TIME_TMTB_0_0, detail

recode TIME_TMTB_0_0 (1990 = .)

**** Trail Making Test Part B log transformation***

generate ln_TMTB_0_0 = ln(TIME_TMTB_0_0)

tabstat ln_TMTB_0_0, stats (n mean median sd min max)

summarize ln_TMTB_0_0, detail

**** Score fluid intelligence***

clonevar cog_fi = n_20191_0_0

tab1 cog_fi, freq m

histogram cog_fi, freq

summarize cog_fi, detail

tabstat cog_fi, stats (n mean median sd min max)

**** Spearman Correlation***

spearman townsend coast_0 green3_0_0 log_vm6_0_1 ln_TMTB_0_0 cog_fi age_0_0 imp_years_of_education, star(0.01) bon

spearman townsend coast_0 green3_0_0 log_vm6_0_1 ln_TMTB_0_0 cog_fi age_0_0 imp_years_of_education, stats(rho p) bon

**** Model***

sem (townsend -> log_vm6_0_1, ) (townsend -> ln_TMTB_0_0, ) (townsend -> cog_fi, ) (coast_0 -> log_vm6_0_1, ) (coast_0 -> ln_TMTB_0_0, ) (coast_0 -> cog_fi, ) (age_0_0 -> log_vm6_0_1, ) (age_0_0 -> ln_TMTB_0_0, ) (age_0_0 -> cog_fi, ) (imp_years_of_education -> townsend, ) (imp_years_of_education -> coast_0, ) (imp_years_of_education -> log_vm6_0_1, ) (imp_years_of_education -> ln_TMTB_0_0, ) (imp_years_of_education -> cog_fi, ) (imp_years_of_education -> green3_0_0, ) (green3_0_0 -> log_vm6_0_1, ) (green3_0_0 -> ln_TMTB_0_0, ) (green3_0_0 -> cog_fi, ), method(mlmv) standardized cov( e.townsend*e.coast_0 e.townsend*e.green3_0_0 e.log_vm6_0_1*e.ln_TMTB_0_0 e.log_vm6_0_1*e.cog_fi e.ln_TMTB_0_0*e.cog_fi imp_years_of_education*age_0_0 e.green3_0_0*e.coast_0) nocapslatent

estat gof, stats(all)
